# Cryoballoon Ablation of the Left Common Pulmonary Vein Using a Size‐Adjustable Cryoballoon: A Comparative Study

**DOI:** 10.1002/joa3.70208

**Published:** 2025-10-17

**Authors:** Hirofumi Arai, Yasuteru Yamauchi, Yuichiro Sagawa, Kazuya Murata, Atsuhito Oda, Yumi Yasui, Junichi Kishaba, Hideki Arima, Shinsuke Miyazaki, Tetsuo Sasano

**Affiliations:** ^1^ Department of Cardiology Japan Red Cross Yokohama City Bay Hospital Yokohama Kanagawa Japan; ^2^ Department of Cardiovascular Medicine Institute of Science Tokyo Bunkyo Tokyo Japan

**Keywords:** atrial fibrillation, catheter ablation, cryoablation, pulmonary veins, treatment outcome

## Abstract

**Background:**

The efficacy of cryoballoon ablation (CBA) using a 28‐mm or 31‐mm cryoballoon for isolating the left common pulmonary vein (LCPV) remains poorly established. We aimed to evaluate procedural outcomes and long‐term follow‐up data of CBA for the LCPV using either POLARx with a fixed 28‐mm cryoballoon or POLARx FIT with a size‐adjustable 28‐mm or 31‐mm cryoballoon.

**Methods:**

Patients with LCPV who underwent CBA for atrial fibrillation using POLARx or POLARx FIT between January 2022 and April 2024 were retrospectively analyzed. Procedural outcomes and long‐term follow‐up data were compared between the POLARx and POLARx FIT groups.

**Results:**

Fifty‐one patients (32 males [62.7%]; mean age, 66 ± 11.6 years) were analyzed. The POLARx group included 23 patients, and the POLARx FIT group included 28 patients. First‐freeze pulmonary vein isolation (PVI) success was 1 (4.3%) versus 8 (28.6%) (*p* = 0.03), and radiofrequency touch‐up ablation was required in 2 (8.7%) versus 0 patients (*p* = 0.2) in the POLARx and POLARx FIT groups, respectively. Nadir temperature was −53.6° ± 4.7°C versus −54.7° ± 5.6°C (*p* = 0.45); the number of applications was 3.2 ± 1 versus 2.3 ± 0.9 (*p* < 0.01); and total freezing time was 520.9 ± 164 s versus 377.7 ± 129.9 s (*p* < 0.01) for the POLARx and POLARx FIT groups, respectively. A single gastric hypomotility case was observed in the POLARx FIT group. The 1‐year arrhythmia‐free survival rates were 81.8% and 78.7% for the POLARx and POLARx FIT groups, respectively (*p* = 0.96).

**Conclusions:**

POLARx FIT was useful for LCPV isolation, with a higher first‐freeze PVI success rate, fewer applications, and shorter total freezing time compared to POLARx.

## Graphical abstract

The POLARx FIT demonstrated higher first‐freeze PVI success, with fewer applications and shorter freezing time than the POLARx, while both systems achieved approximately 80% arrhythmia‐free survival at 1 year.

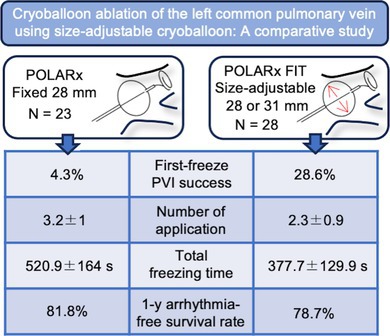

## 1 Introduction

Cryoballoon ablation (CBA) is an established treatment for atrial fibrillation (AF), and the efficacy of the novel POLARx cryoballoon (Boston Scientific, Marlborough, MA) is comparable to the conventional Arctic Front Advance (AFA) cryoballoon (Medtronic, Minneapolis, MN) [1, 2]. The original POLARx had a fixed 28‐mm cryoballoon; however, the newer POLARx FIT (Boston Scientific), a size‐adjustable cryoballoon offering either a 28‐mm or 31‐mm option, was subsequently introduced. The POLARx FIT demonstrates a high acute success rate in pulmonary vein (PV) isolation (PVI), and the 31‐mm cryoballoon enables more proximal occlusion of the PV compared to its predecessor [3]. Furthermore, the occlusion achieved with the 31‐mm cryoballoon is superior to that of the 28‐mm cryoballoon, including in the case of the left common PV (LCPV) [4].

CBA for the LCPV using the AFA is effective, with acute success and long‐term durability of PVI comparable to procedures without LCPV [5, 6, 7]. The efficacy of CBA for LCPV is comparable to that of radiofrequency catheter ablation [8, 9, 10]. However, CBA for LCPV remains challenging and controversial, as LCPV may be a risk factor for AF recurrence following ablation with the AFA [11, 12]. The POLARx FIT with the 31‐mm cryoballoon may improve the outcomes of LCPV ablation due to its superior occlusion; however, data on CBA for the LCPV using the POLARx or the POLARx FIT are limited. This study aimed to evaluate the procedural outcomes and long‐term follow‐up data of LCPV CBA using the POLARx with a fixed 28‐mm balloon or the POLARx FIT with a size‐adjustable 28‐mm or 31‐mm balloon.

## 2 Methods

### 2.1 Study Population and Preoperative Management

**FIGURE 1:**
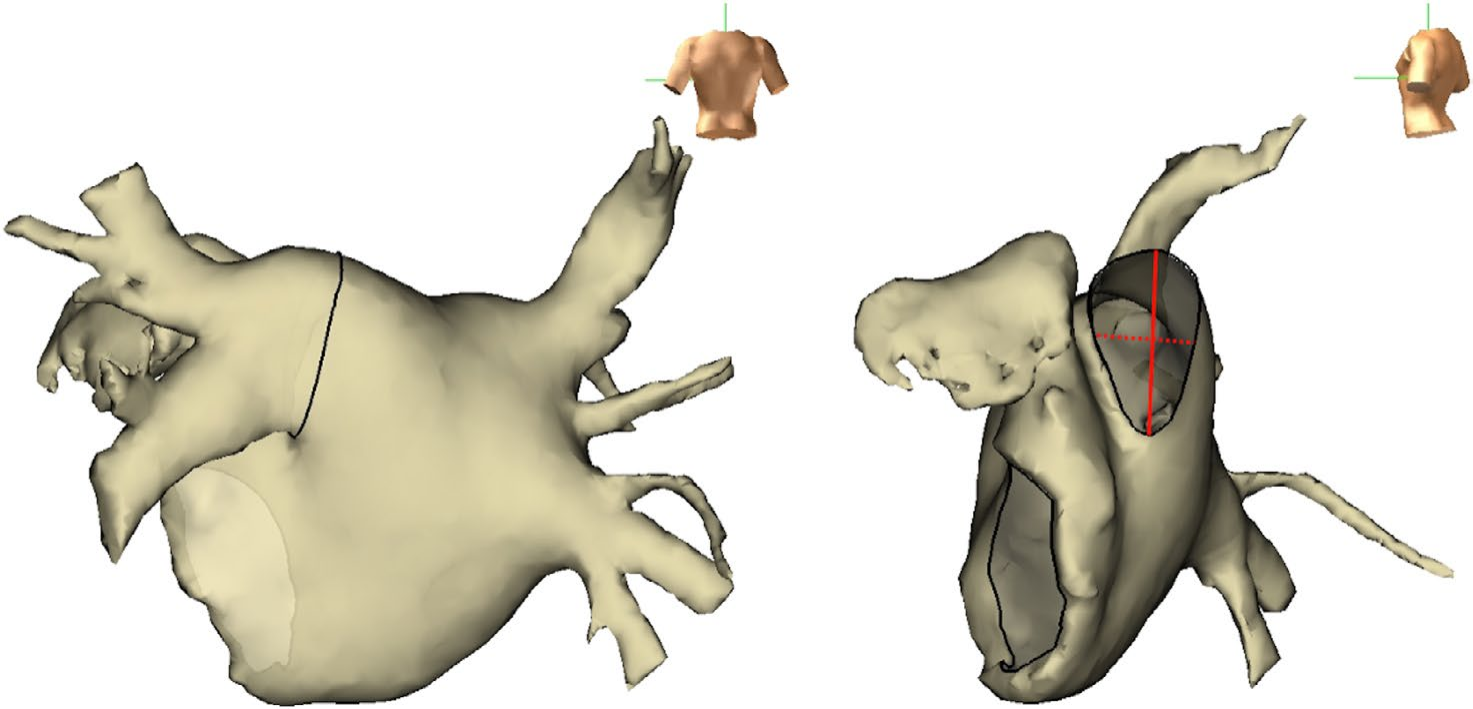
Measurements of the left common pulmonary vein. Three‐dimensional images of the left atrium and pulmonary veins were obtained via contrast‐enhanced computed tomography. The left common pulmonary vein ostium was identified (black line), and the maximum (red solid line) and minimum (red dotted line) diameters of the ostium were measured. The ovality index was calculated using the following formula: 2 × (maximal diameter – minimal diameter)/(maximal diameter + minimal diameter).

Patients with LCPV were enrolled among those who underwent an index PVI procedure using either POLARx or POLARx FIT between January 2022 and April 2024 at the Japan Red Cross Yokohama City Bay Hospital. The POLARx system was used from January 2022 to March 2023, and the POLARx FIT from April 2023 to April 2024. The indication for PVI followed the JCS/JHRS 2019 Guideline on Non‐Pharmacotherapy of Cardiac Arrhythmias [13]. Anticoagulation therapy continued for over 3 weeks before the procedure, and antiarrhythmic medications were discontinued for more than five half‐lives before ablation. Left atrial appendage thrombus was excluded, and the PV anatomy was evaluated using prone position contrast‐enhanced computed tomography (CT). Three‐dimensional (3D) images of the PVs and left atrium were reconstructed from CT findings. A left PV was defined as an LCPV when it had a distance of ≥ 5 mm from the left atrial virtual border to the bifurcation of the superior and inferior branches [5, 14]. For patients with LCPV, the maximal and minimal diameters of the LCPV ostium and the length of the left common trunk were measured using 3D imaging, and the ostium area and ovality index [2 × (maximal diameter – minimal diameter)/(maximal diameter + minimal diameter)] were calculated (Figure 1). This study was approved by the Research Ethics Committee of Japan Red Cross Yokohama City Bay Hospital.

### 2.2 Cryoballoon Ablation for the LCPV

Total intravenous anesthesia was administered using propofol, and the bispectral index was maintained between 40 and 60 throughout the procedure. An i‐gel (Intersurgical, Wokingham, Berkshire, UK) supraglottic airway device was placed for mechanical ventilation, and an esophageal luminal temperature catheter was inserted. Arterial blood pressure was monitored via a right radial or femoral artery catheter. Activated clotting time was maintained above 350 s using a continuous infusion of unfractionated heparin. A coronary sinus catheter was inserted via the right jugular vein. Two SL‐0 sheaths (Abbott, Chicago, IL) were inserted into the right femoral vein, and a transseptal puncture was performed with a radiofrequency (RF) needle (Boston Scientific) under intracardiac echocardiography guidance. 3D electro‐anatomical mapping (EAM) was performed using an Advisor HD Grid mapping catheter (Abbott) or an Octaray mapping catheter (Johnson and Johnson, New Brunswick, NJ) with the EnSite (Abbott) or CARTO (Johnson and Johnson) 3D EAM systems. A POLAR sheath (Boston Scientific) was introduced into the left atrium, and a POLARmap circular mapping catheter was advanced into the LCPV. Subsequently, CBA of the LCPV was performed using either the POLARx or POLARx FIT. Occlusion of the LCPV ostium was attempted, and freezing was initiated upon confirmation of complete occlusion. The initial POLARx FIT freezing used a 31 mm balloon, and subsequent balloon sizes were selected at the operator's discretion. CBA with the POLARx was performed using a 28 mm balloon. When complete occlusion of the LCPV ostium was not feasible, non‐occlusive separate freezing was performed by targeting the superior and inferior portions of the LCPV ostium separately [15]. If unsuccessful, a sequential ablation approach was applied to the superior and inferior distal branches [5]. When the PV potential remained, additional freezing was applied according to the sequence of the PV potential in the POLARmap. The standard freezing time was 180 s, but it was extended to 210 or 240 s depending on the time to isolation (TTI). Additional CBA after achieving PVI was performed when the TTI was over 90 s or unknown. Freezing was terminated and balloon deflated using the double stop technique when the esophageal temperature reached 15°C. Completion of LCPV isolation was confirmed by eliminating the PV potentials and 3D EAM after PVI. Radiofrequency (RF) touch‐up catheter ablation was considered when PVI was not achieved within four times freezing or reconnection of the PVI was seen in the 3D EAM after the CBA. For patients with paroxysmal AF (PAF), only PVI was performed. For patients with non‐PAF, additional posterior wall isolation with roof line CBA and floor line RF ablation was conducted. The LCPV ostium complete occlusion rate, the first‐freeze PVI success rate, RF touch‐up ablation rate, mean nadir temperature, number of applications, total freezing time, rate of the esophageal temperature drop, and incidence of gastroesophageal complications were assessed between the POLARx and the POLARx FIT systems.

### 2.3 Follow‐Up

All patients were followed up during outpatient visits within 1 month after the procedure and every 1–3 months thereafter. A 12‐lead electrocardiogram (ECG) was performed at all outpatient visits, and 24‐h or 7‐day Holter monitoring was conducted at 3 and 12 months. Patients were followed up for at least 1 year. Arrhythmic recurrence was defined as atrial arrhythmia lasting for > 30 s on Holter monitoring or detected by a 12‐lead ECG after a 90‐day blanking period. Antiarrhythmic drugs were administered at the discretion of the treating physician.

### 2.4 Statistical Analysis

Categorical variables are presented as numbers (%). Continuous variables with normal distribution are described as the mean ± standard deviation, while those with continuous variables with non‐normal distribution are described as the median [25th, 75th percentiles]. Fisher's exact test was used to analyze categorical variables. The unpaired t‐test was used for continuous variables with a normal distribution, and the Mann–Whitney *U*‐test was used for those with a non‐normal distribution. Arrhythmia‐free survival was assessed using the Kaplan–Meier method. Statistical significance was set at *p* < 0.05. EZR [16] (Saitama Medical Center, Jichi Medical University, Saitama, Japan), a graphical user interface for R software (R Foundation for Statistical Computing, Vienna, Austria), was used for the analysis.

## 3 Results

### 3.1 Baseline Characteristics and CT Findings

A total of 51 patients with LCPV (mean age, 66 ± 11.6) were analyzed (Table 1). Of these, 32 (62.7%) were male, 33 (64.7%) patients had PAF, and 18 (35.3%) patients had non‐PAF. The POLARx group included 23 patients (45.1%), while the POLARx FIT group included 28 (54.9%). No significant differences in baseline characteristics were observed between the groups. Left atrial volume, LCPV maximum and minimum diameters, length of the left common trunk, LCPV ostium area, and ovality index did not differ significantly between the two groups (Table 2).

**TABLE 1:** Baseline characteristics.

	All	POLARx	POLARx FIT	*p*
*n*	51	23	28	
Male, *n* (%)	32 (62.7)	13 (56.5)	19 (67.9)	0.56
Age, years	66 ± 11.6	67.1 ± 9.9	65.2 ± 13	0.56
BMI, kg/m^2^	23.7 ± 5	22.9 ± 4.9	24.3 ± 5	0.3
Past medical history				
Heart failure, *n* (%)	9 (17.6)	4 (17.4)	5 (17.9)	> 0.99
Hypertension, *n* (%)	28 (54.9)	11 (47.8)	17 (60.7)	0.41
Diabetes mellitus, *n* (%)	2 (3.9)	0 (0)	2 (7.1)	0.5
Stroke/TIA, *n* (%)	4 (7.8)	2 (8.7)	2 (7.1)	> 0.99
CHADS_2_ score	1.2 ± 1	1.1 ± 0.9	1.3 ± 1	0.39
CHA_2_DS_2_‐VAS_C_ score (%)	2.2 ± 1.5	2.2 ± 1.3	2.2 ± 1.7	0.93
Echocardiography				
LVEF, %	64.6 ± 8.8	64.5 ± 9.2	64.7 ± 8.6	0.94
LAD, mm	41.9 ± 8.1	41.1 ± 7.7	42.7 ± 8.6	0.5
Type of atrial fibrillation				
PAF, *n* (%)	33 (64.7)	15 (65.2)	18 (64.3)	> 0.99
Non‐PAF, *n* (%)	18 (35.3)	8 (34.8)	10 (35.7)	> 0.99

Abbreviations: BMI, body mass index; LAD, left atrial dimension; LVEF, left ventricular ejection fraction; PAF, paroxysmal atrial fibrillation; TIA, transient ischemic attack.

**TABLE 2:** CT findings.

	All	POLARx	POLARx FIT	*p*
Left atrial volume, mL	123.8 ± 43	121.7 ± 48	125.5 ± 39.2	0.76
LCPV max diameter, mm	32.4 ± 6.3	31.7 ± 6.3	32.9 ± 5.7	0.49
LCPV min diameter, mm	22.9 ± 5.8	21.9 ± 4.4	23.7 ± 6.6	0.27
Length of left common trunk, mm	10.8 ± 3.5	11.2 ± 3.6	10.4 ± 3.5	0.48
LCPV ostium area, mm^2^	608.6 ± 269.8	570.5 ± 209.8	638.6 ± 309.4	0.38
Ovality index	0.35 ± 0.17	0.37 ± 0.18	0.34 ± 0.16	0.64

Abbreviations: LCPV, left common pulmonary vein; max, maximum; min, minimum.

### 3.2 Procedural Results

**FIGURE 2:**
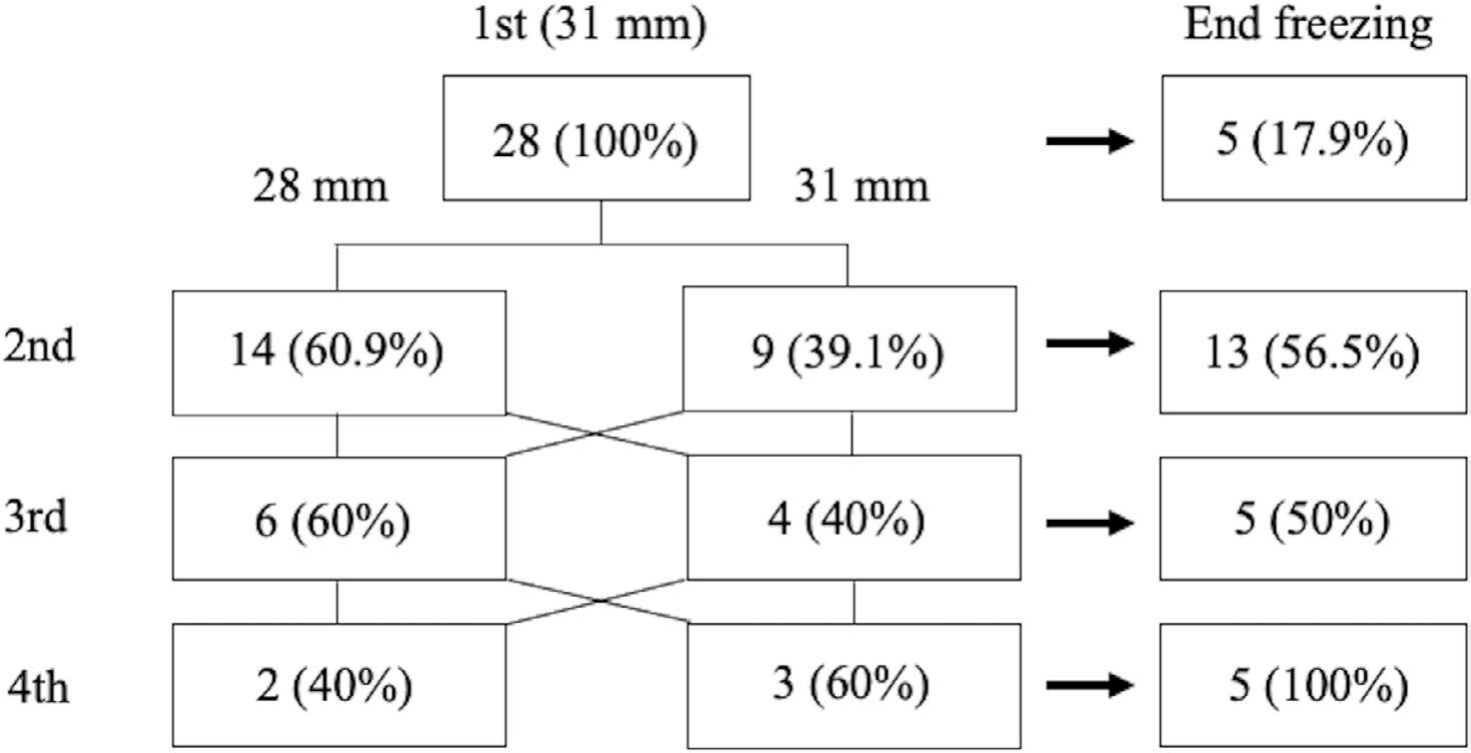
Balloon size selection of the POLARx FIT. The first application of the POLARx FIT was performed with a 31‐mm cryoballoon in all 28 patients. Five of the eight patients who achieved complete occlusion completed the procedure after the first freezing; the remaining three patients underwent additional freezing. The balloon size for the subsequent procedures was determined at the discretion of the operator. Among the 23 patients who underwent second freezing, a 28‐mm cryoballoon was used for 14 (60.9%) patients; a 31‐mm cryoballoon was used in 9 (39.1%) patients; and 13 (56.5%) patients ended freezing after the second freezing. Thereafter, a 28‐mm cryoballoon was used for 6 (60%) patients; a 31‐mm cryoballoon was used for 4 (40%) patients for the third freezing; a 28‐mm cryoballoon was used for 2 (40%) patients; and a 31‐mm cryoballoon was used for 3 (60%) patients for the fourth freezing.

Complete occlusion of the LCPV ostium was achieved in 1 of 23 patients (4.3%) in the POLARx group and 9 of 28 patients (32.1%) in the POLARx FIT group (*p* = 0.02). Among them, first‐freeze PVI success was achieved in 1 (4.3%) patient in the POLARx group and 8 (28.6%) patients in the POLARx FIT group (*p* = 0.03) (Table 3). The rate of RF touch‐up ablation did not differ significantly between groups, although two patients (8.7%) in the POLARx group required RF touch‐up ablation because of the PV reconnection after the CBA. Conversely, no patients needed RF touch‐up ablation in the POLARx FIT group. While nadir temperatures did not differ significantly, the number of applications was lower (3.2 ± 1 vs. 2.3 ± 0.9, *p* < 0.01), and the total freezing time was shorter (520.9 ± 164 s vs. 377.7 ± 129.9 s, *p* < 0.01) in the POLARx FIT group. One gastric hypomotility event occurred in the POLARx FIT group; however, the incidence of gastric hypomotility did not differ significantly between the two groups. Among the eight patients who achieved first‐freeze PVI success using the POLARx FIT, five completed the CBA at the first freeze and the remaining three underwent additional CBA after achieving PVI because the TTI was over 90 s or unknown (Figure 2). The balloon size for the POLARx FIT group after the first freezing was chosen, focusing on not only occlusion state but also the balloon attachment to the PV tissue according to the operator's discretion.

**TABLE 3:** Procedure results.

	All	POLARx	POLARx FIT	*p*
LCPV ostium complete occlusion, *n* (%)	10 (19.6)	1 (4.3)	9 (32.1)	0.02
First‐freeze PVI success, *n* (%)	9 (17.6)	1 (4.3)	8 (28.6)	0.03
RF touch‐up ablation, *n* (%)	2 (3.9)	2 (8.7)	0 (0)	0.2
Nadir temperature, °C	−54.2 ± 5.1	−53.6 ± 4.7	−54.7 ± 5.6	0.45
Number of applications, times	2.7 ± 1.1	3.2 ± 1	2.3 ± 0.9	< 0.01
Total freezing time, s	442.3 ± 161.7	520.9 ± 164	377.7 ± 129.9	< 0.01
Esophageal temperature drop, *n* (%)	23 (45.1)	12 (52.2)	11 (39.3)	0.41
Gastric hypomotility, *n* (%)	1 (2)	0 (0)	1 (3.6)	> 0.99

Abbreviations: LCPV, left common pulmonary vein; PVI, pulmonary vein isolation; RF, radiofrequency.

### 3.3 Achievement of Complete Occlusion by the POLARx FIT and Anatomical Features of the LCPV

Complete occlusion of the LCPV ostium was achieved in nine patients in the POLARx FIT group. These patients tended to have a smaller LCPV maximal diameter (30.3 ± 3.4 mm vs. 34.1 ± 6.2 mm, *p* = 0.09), smaller LCPV minimal diameter (21.6 ± 4.1 mm vs. 24.7 ± 7.4 mm, *p* = 0.25), smaller LCPV ostium area (507.7 ± 134.7 mm^2^ vs. 700.5 ± 350.5 mm^2^, *p* = 0.13), and longer left common trunk (11.9 ± 3.6 mm vs. 9.7 ± 3.3 mm, *p* = 0.14) compared to the patients who could not achieve complete occlusion; however, there were no significant differences. The ovality index was comparable (0.34 ± 0.13 mm vs. 0.34 ± 0.18 mm, *p* = 0.96) between the patients with or without complete occlusion.

### 3.4 Long‐Term Follow‐Up

**FIGURE 3:**
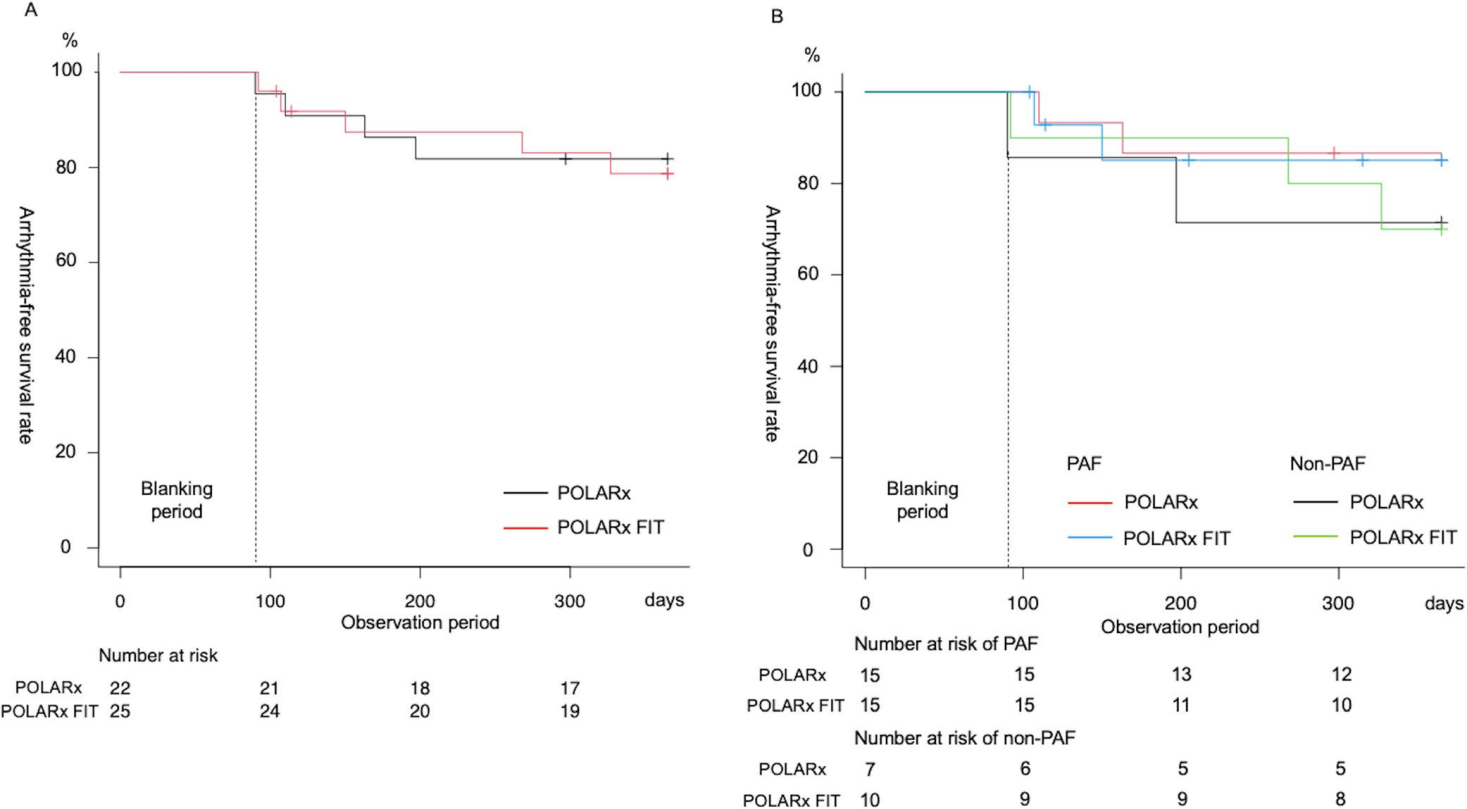
One‐year arrhythmia‐free survival rate. The 1‐year arrhythmia‐free survival rate of all patients of POLARx was 81.8% (95% CI: 58.5%–92.8%) and that of the POLARx FIT was 78.7% (95% CI: 56.1%–90.6%), *p* = 0.92 (A). Among the paroxysmal atrial fibrillation (PAF) patients, the 1‐year arrhythmia‐free survival rate of the POLARx was 86.7% (95% CI: 56.4%–96.5%) and that of the POLARx FIT was 85.1% (95% CI: 52.3%–96.1%). Among the non‐PAF patients, the 1‐year arrhythmia‐free survival rates of the POLARx were 71.4% (95% CI: 25.8%–92%) and that of the POLARx FIT was 70% (95% CI: 32.9%–89.2%) (B).

The 1‐year arrhythmia‐free survival rate of the POLARx group was 81.8% and that of the POLARx FIT group was 78.7% (*p* = 0.96) (Figure 3). Follow‐up was interrupted before the 90‐day blanking period in one patient from the POLARx group and in three patients from the POLARx FIT group. Median follow‐up duration, arrhythmia recurrence, number of patients who underwent re‐ablation, LCPV reconnection, and antiarrhythmic drug use did not differ significantly between the groups (Table 4).

**TABLE 4:** Long‐term follow‐up data.

	All	POLARx	POLARx FIT	*p*
Patients followed after blanking period, *n* (%)	47	22	25	
Follow‐up period, *n* (%)	400 [232.5, 521]	427 [332, 684.5]	382.5 [141, 446]	0.07
Arrhythmia recurrence, *n* (%)	11 (23.4)	6 (27.3)	5 (20)	0.73
Re‐ablation, *n* (%)	9 (19.1)	5 (22.7)	4 (16)	0.73
Reconnection of LCPV, *n* (%)	3 (6.4)	2 (9.1)	1 (4)	> 0.99
Antiarrhythmic drug, *n* (%)	8 (17)	5 (22.7)	3 (12)	0.45

Abbreviation: LCPV, left common pulmonary vein.

## 4 Discussion

POLARx FIT demonstrated a higher first‐freeze PVI success rate, fewer applications, and a shorter total freezing time for LCPV isolation compared with POLARx. The incidence of gastric hypomotility did not differ significantly between groups. The 1‐year arrhythmia‐free survival rate was similar between the two groups, reaching approximately 80%.

In a previous report from the multicenter registry of the POLARx FIT, 12 LCPVs were included; 8 (66.7%) achieved one‐shot isolation, and 2 (16.7%) required RF touch‐up ablation [3]. Although the one‐shot isolation rate was higher, the RF touch‐up ablation rate was also higher than that in the present study. Precise assessment was limited because the previous report did not include data on PV ostium diameter, length of the left common trunk, or ovality index, which may have influenced CBA outcomes. Previous reports of the CBA for the LCPV using AFA show that patients treated with single shot freeze have a longer left common trunk and smaller ostial area compared to the patients needing segmental freeze [6]. The patients that achieved complete LCPV ostium occlusion by the POLARx FIT in our study had similar tendencies, but there were no significant differences compared to the patients who did not achieve complete occlusion. The sample size may have been too small to determine a statistically significant difference. However, the fact remained that the 31‐mm cryoballoon could achieve PVI by the first freeze even for the LCPV. The 31‐mm cryoballoon could occlude the PV more proximally than the 28‐mm cryoballoon [3], and create a large lesion in the antrum [17, 18]. These characteristics also apply to LCPV CBA and may have contributed to the higher first‐freeze PVI success rate, fewer applications, and shorter total freezing time observed in this study. One patient with complete occlusion after the first attempt using POLARx FIT could not achieve PVI with the first freezing, and the second freezing achieved PVI. The same phenomenon using the POLARx or the POLARx FIT has been reported, with large PV diameter being one of the risk factors [19]. The LCPV was larger than normal PV, so that some cases might not achieve PVI even though complete occlusion was achieved.

Isolation at the proximal side of the LCPV is important due to the arrhythmogenic potential of its common trunk [20]. A sequential ablation approach occluding each superior and inferior branch of the LCPV has been reported when antral occlusion could not be achieved [5]; however, residual PV potentials at the proximal side of the LCPV remained a concern with this method [21]. Non‐occlusive separate freezing [15]—targeting the superior and inferior portions of the PV ostium without full occlusion—was applied before the sequential ablation to prevent distal isolation; however, this method required at least two applications to achieve PVI. The 31‐mm cryoballoon may be a favorable option for LCPV because it can achieve antral occlusion more easily than the 28‐mm cryoballoon and can decrease the number of applications. However, additional CBA should be considered even if PVI is achieved with the first freeze, due to the importance of ensuring antral isolation of the LCPV.

Eight patients achieved PVI with the first freeze; however, three required additional CBA because of the TTI > 90 s or unknown, and nearly half of the subsequent applications involved the 28‐mm cryoballoon. Most additional CBA procedures at the PV antrum were conducted in a non‐occlusive state [22]. Cryoballoon compliance and myocardial contact should be assessed in that situation because the cryoballoon contact may influence the PV reconnection [23]. The 31‐mm cryoballoon exhibits lower compliance than the 28‐mm version due to its higher internal gas pressure. This might cause the 31‐mm cryoballoon to have a lower balloon contact area than the 28‐mm cryoballoon in the non‐occlusive state. The ability to adjust balloon size according to the purpose of CBA is an advantage of the POLARx FIT system. However, there were no definitive selection criteria for the balloon size after the first freezing, and this was chosen according to the operator's discretion. That might influence the results of the POLARx FIT group.

One case of gastric hypomotility occurred in the POLARx FIT group; however, no significant difference in complication rates was observed between the two groups. The incidence of esophageal temperature drop was also not significantly different between groups. The patient with gastric hypomotility did not require hospitalization, was managed through an outpatient visit with medication therapy, and fully recovered within 3 months following the procedure. The patient with gastric hypomotility had non‐PAF; required freezing three times, with 31‐mm, 31‐mm, 28‐mm cryoballoons for the LCPV; and underwent roofline CBA with a 28‐mm cryoballoon and floor line RF ablation. A previous multicenter registry study of the POLARx FIT reported no atrio‐esophageal fistula but did not mention gastric hypomotility [3]. A previous study of non‐occlusive separate freezing for the large PV reported that patients with gastric hypomotility using the POLARx had significantly longer freezing times than patients without gastric hypomotility, so that longer and frequent freezing may be a risk factor for gastric hypomotility [15]. Additionally, in the report of the relation of the roof line ablation using the POLARx and gastric hypomotility, additional roof line ablation and a shorter distance from the esophagus to the midpoint of the vertebral body at the level of the lowest left atrial roof were reported as risk factors for gastric hypomotility [24]. The reported cutoff value of the distance between the esophagus and the midpoint of the vertebral body is 20 mm. Our patient with gastric hypomotility had a distance of 15 mm. Cryoballoon freezing for the LCPV with a 31‐mm balloon might influence gastric hypomotility; however, the patient had other risk factors for gastric hypomotility such as relatively long freezing, cryoballoon roof line ablation, and a short distance between the esophagus and vertebral body. It might be difficult to evaluate gastric hypomotility as a specific complication of the POLARx FIT. The incidence of gastric hypomotility was too small to assess, and resulted from differences between the POLARx and POLARx FIT in this study.

The 1‐year arrhythmia‐free survival rate was approximately 80%, with no significant difference observed between the POLARx and the POLARx FIT. The arrhythmia‐free survival of the AF patients with LCPV treated by the AFA ranges from 45.6% to 74% [5, 6, 7, 8, 9, 10, 11], and the presence of LCPV might be a risk factor for AF recurrence [11, 12]. Freezing in a non‐occlusive state, such as during left atrial posterior wall CBA, is sometimes needed for LCPV CBA. The nadir temperature of the POLARx was significantly lower than that of the AFA, both during PVI with pulmonary vein occlusion and during left atrial posterior wall isolation using non‐occlusive CBA [25]. The lower nadir temperature might contribute to the relatively high arrhythmia‐free survival rate observed with POLARx and POLARx FIT in the present study. This rate is considered acceptable for the treatment of AF. Both POLARx and POLARx FIT may be effective for PVI of the LCPV, even over the long term.

Our study had some limitations. First, this was a single‐center study, which limits the generalizability of the results. However, selection bias was minimal, as all patients with AF underwent CBA at the same institution. Second, the follow‐up period for POLARx tended to be longer than that for POLARx FIT because POLARx was introduced earlier. The median follow‐up period was not significantly different between the two groups, which may have influenced the long‐term follow‐up data. The earlier introduction of the POLARx may also influence the learning curve and cause higher success rates of the POLARx FIT. However, the influence of the learning curve might not be as great because AFA has been frequently used before this study and the procedure using the POLARx or the POLARx FIT resembles the procedure using AFA. Third, three patients in the POLARx FIT group and only one patient in the POLARx group discontinued follow‐up. One patient moved to another hospital and the other two patients defaulted on follow‐up. The difference in the number of patients that discontinued follow‐up might influence the long‐term follow‐up data. Fourth, arrhythmia recurrence was confirmed by 12‐lead ECG at every outpatient visit and 24‐h or 7‐day Holter monitoring at 3 and 12 months. This approach may have led to underestimation of arrhythmia recurrence and overestimation of arrhythmia‐free survival. Finally, while antiarrhythmic drug use did not significantly differ during long‐term follow‐up, it was subject to physician discretion. This may have influenced arrhythmia recurrence.

## 5 Conclusions

The POLARx FIT may be useful for LCPV isolation because of its higher first‐freeze PVI success rate, fewer applications, and shorter total freezing time compared to the POLARx. Both systems showed approximately 80% arrhythmia‐free survival rate at 1 year.

## Ethics Statement

This study was approved by the Research Ethics Committee of Japan Red Cross Yokohama City Bay Hospital. The approval number is 2024‐13.

## Consent

Informed consent was obtained in the form of an opt‐out.

## Conflicts of Interest

The authors declare no conflicts of interest.

## Data Availability

The data underlying this article will be shared on reasonable request to the corresponding author.
